# A longitudinal study of associations between HIV‐related stigma, recent violence and depression among women living with HIV in a Canadian cohort study

**DOI:** 10.1002/jia2.25341

**Published:** 2019-07-22

**Authors:** Carmen H Logie, Natania Marcus, Ying Wang, Angela Kaida, Patricia O'Campo, Uzma Ahmed, Nadia O'Brien, Valerie Nicholson, Tracey Conway, Alexandra de Pokomandy, Mylène Fernet, Mona Loutfy, the CHIWOS Research Team, Aranka Anema, Denise Becker, Lori Brotto, Allison Carter, Claudette Cardinal, Guillaume Colley, Erin Ding, Janice Duddy, Nada Gataric, Robert S Hogg, Terry Howard, Shahab Jabbari, Evin Jones, Mary Kestler, Andrea Langlois, Viviane Lima, Elisa Lloyd‐Smith, Melissa Medjuck, Cari Miller, Deborah Money, Gina Ogilvie, Sophie Patterson, Neora Pick, Eric Roth, Kate Salters, Margarite Sanchez, Jacquie Sas, Paul Sereda, Marcie Summers, Christina Tom, Lu Wang, Kath Webster, Wendy Zhang, Rahma Abdul‐Noor, Jonathan Angel, Fatimatou Barry, Greta Bauer, Kerrigan Beaver, Anita Benoit, Breklyn Bertozzi, Sheila Borton, Tammy Bourque, Jason Brophy, Ann Burchell, Allison Carlson, Lynne Cioppa, Jeffrey Cohen, Curtis Cooper, Jasmine Cotnam, Janette Cousineau, Brenda Gagnier, Claudine Gasingirwa, Saara Greene, Trevor Hart, Shazia Islam, Logan Kennedy, Desiree Kerr, Maxime Kiboyogo, Gladys Kwaramba, Lynne Leonard, Johanna Lewis, Shari Margolese, Marvelous Muchenje, Mary, Ndung'u, Kelly O'Brien, Charlene Ouellette, Jeff Powis, Corinna Quan, Janet Raboud, Anita Rachlis, Edward Ralph, Sean Rourke, Sergio Rueda, Roger Sandre, Fiona Smaill, Stephanie Smith, Tsitsi Tigere, Wangari Tharao, Sharon Walmsley, Wendy Wobeser, Jessica Yee, Mark Yudin, Dada Mamvula Bakombo, Jean‐Guy Baril, Nora Butler Burke, Pierrette Clément, Janice Dayle, Danièle Dubuc, Danielle Groleau, Aurélie Hot, Marina Klein, Carrie Martin, Lyne Massie, Brigitte Ménard, Nadia O'Brien, Joanne Otis, Doris Peltier, Alie Pierre, Karène Proulx‐Boucher, Danielle Rouleau, Édénia Savoie, Cécile Tremblay, Benoit Trottier, Sylvie Trottier, Christos Tsoukas, Jacqueline Gahagan, Catherine Hankins, Renee Masching, Susanna Ogunnaike‐Cooke

**Affiliations:** ^1^ Factor‐Inwentash Faculty of Social Work University of Toronto Toronto Ontario Canada; ^2^ Women's College Research Institute Women's College Hospital Toronto Ontario Canada; ^3^ Department of Applied Psychology and Human Development University of Toronto Toronto Ontario Canada; ^4^ Faculty of Health Sciences Simon Fraser University Vancouver British Columbia Canada; ^5^ St. Michael's Hospital Toronto Ontario Canada; ^6^ Dalla Lana School of Public Health University of Toronto Toronto Ontario Canada; ^7^ Department of Family Medicine McGill University Montreal Quebec Canada; ^8^ Chronic Viral Illness Service McGill University Health Centre Montreal Quebec Canada; ^9^ Department of Sexology Université du Québec à Montréal Montreal Quebec Canada; ^10^ Department of Medicine University of Toronto Toronto Ontario Canada

**Keywords:** women, violence, HIV, stigma, gender, depression

## Abstract

**Introduction:**

Women living with HIV (WLHIV) experience stigma and elevated exposure to violence in comparison with HIV‐negative women. We examined the mediating role of experiencing recent violence in the relationship between stigma and depression among WLHIV in Canada.

**Methods:**

We conducted a cohort study with WLHIV in three Canadian provinces. Recent violence was assessed through self‐reported experiences of control, physical, sexual or verbal abuse in the past three months. At Time 1 (2013‐2015) three forms of stigma were assessed (HIV‐related, racial, gender) and at Time 2 (2015‐2017) only HIV‐related stigma was assessed. We conducted structural equation modelling (SEM) using the maximum likelihood estimation method with Time 1 data to identify direct and indirect effects of gender discrimination, racial discrimination and HIV‐related stigma on depression via recent violence. We then conducted mixed effects regression and SEM using Time 1 and Time 2 data to examine associations between HIV‐related stigma, recent violence and depression.

**Results:**

At Time 1 (n = 1296), the direct path from HIV‐related stigma (direct effect: β = 0.200, *p* < 0.001; indirect effect: β = 0.014, *p* < 0.05) to depression was significant; recent violence accounted for 6.5% of the total effect. Gender discrimination had a significant direct and indirect effect on depression (direct effect: β = 0.167, *p* < 0.001; indirect effect: β = 0.050, *p* < 0.001); recent violence explained 23.15% of the total effect. Including Time 1 and Time 2 data (n = 1161), mixed‐effects regression results indicate a positive relationship over time between HIV‐related stigma and depression (Acoef: 0.04, 95% CI: 0.03, 0.06, *p* < 0.001), and recent violence and depression (Acoef: 1.95, 95% CI: 0.29, 4.42, *p* < 0.05), controlling for socio‐demographics. There was a significant interaction between HIV‐related stigma and recent violence with depression (Acoef: 0.04, 95% CI: 0.01, 0.07, *p* < 0.05). SEM analyses reveal that HIV‐related stigma had a significant direct and indirect effect on depression over time (direct effect: β = 0.178, *p* < 0.001; indirect effect: β = 0.040, *p* < 0.001); recent violence experiences accounted for 51% of the total effect.

**Conclusions:**

Our findings suggest that HIV‐related stigma is associated with increased experiences of recent violence, and both stigma and violence are associated with increased depression among WLHIV in Canada. There is an urgent need for trauma‐informed stigma interventions to address stigma, discrimination and violence.

## 1 Introduction

Gender‐based violence (GBV) disproportionately impacts and compromises the wellbeing of women and girls across the world 1, 2. A global participatory survey among women living with HIV (WLHIV) found that 89% of those who responded to the GBV section reported having experienced or feared GBV 3. GBV has deleterious impacts on physical and mental health 1, 2. In 2013, the World Health Organization identified violence against women as a public health priority requiring a comprehensive response to enhance prevention and treatment initiatives 4.

GBV is an established risk factor for HIV acquisition 5, 6, 7, 8 and experiencing violence is also prevalent among WLHIV. In Canada, 2015 surveillance data indicate that Canada's HIV prevalence rate is 173 per 100,000 persons 9. Of approximately 63,110 people living with HIV in Canada, women comprised one quarter of HIV cases 9. While Canada does not have national data on intimate partner violence (IPV) among WLHIV, the United States (US) Center for Disease Control reported that the rate of IPV among WLHIV in the US is double the national rate 10.

Violence from both partners and non‐partners has harmful impacts on physical, sexual, reproductive and mental health outcomes 4, 11. Persons who experience violence have reported higher rates of depression, post‐traumatic stress disorder (PTSD), suicide risk and lower self‐esteem 7, 8, 12, 13, 14, 15. While women and men in Canada self‐report similar rates of physical IPV, women are more likely than men to report multiple victimizations, including sexual IPV 16. The body's stress response to violence can have long‐term consequences on the immune system 4, 17, 18, 19. Furthermore, experiencing violence may contribute to the uptake of maladaptive coping mechanisms such as smoking and alcohol abuse, which further contribute to health adversities 4.

Intersectionality is a theoretical framework rooted in Black feminist scholarship 20, 21. Crenshaw (1990) 22 coined the term intersectionality to conceptualize how Black womens’ experiences of discrimination are shaped by the convergence of race, class and gender. Intersectionality examines the interaction between multiple social identities that (re)produce privilege and marginalization 23. Intersectionality is a particularly salient framework for understanding the HIV epidemic, as race, class and gender disparities are structural drivers of HIV 23, 24 An emerging field of research on intersectional stigma assesses the effects of exposure to co‐occurring forms of stigma among WLHIV 25, 26, 27, 28, 29. Previous Canadian research with WLHIV highlights associations between racial discrimination 26, 30, HIV‐related stigma 31, 32, gender discrimination and depression 32. Less known are the associations between multiple forms of stigma and violence exposure among WLHIV.

Syndemics describe the multiple, co‐occurring deleterious psychosocial, structural and environmental factors that negatively impact individual and population level health 33. Syndemics theory provides a framework for understanding the role of violence in producing co‐existing negative social and behavioural outcomes that increase HIV disease progression and vulnerability to other illnesses 34, 35. US studies have examined health consequences of the co‐occurrence of substance abuse, violence, and HIV and AIDS, entitled the “SAVA” syndemic, among WLHIV 36, 37, 38. Tsai 39's recent article called for a shift from exclusively examining individual‐level factors in syndemics research to explore social forces. We respond to this call by examining linkages between stigma and recent violence with elevated depression among WLHIV. Specifically, we used Tsai 39's model of serially causal epidemics that articulates the adverse consequences of accumulating health risks.

There are knowledge gaps regarding longitudinal associations between stigma, violence and depression among WLHIV. This is important to assess, as cross‐sectional research with adolescents living with HIV in South Africa report associations between abuse, HIV‐related stigma and depressive symptoms 40. A recent systematic review of cohort studies examining recent IPV and health outcomes demonstrated bidirectional associations between IPV and depression 15. Yet stigma was not assessed in the review, no included studies were conducted in Canada, and only one study focused on experiences among WLHIV (sex workers in Kenya) 15. We address these knowledge gaps by examining pathways between stigma, violence and depression among WLHIV in Canada's three provinces with the highest number of HIV cases: Ontario (42% of total cases), Quebec (27% of total cases) and British Columbia (16.3% of total cases) 41. At Time 1 we collected information on three forms of stigma (HIV‐related, racial discrimination, gender discrimination) and at Time 2 we only collected information on HIV‐related stigma. Study objectives included: (1) assessing the direct and indirect effects of HIV‐related stigma, racial discrimination and gender discrimination on depression at Time 1, via the mediator of recent violence; and (2) examining associations over time between HIV‐related stigma, recent violence and depression, among WLHIV in a Canadian cohort.

## 2 Methods

### 2.1 Study design and population

We used data from the cross‐sectional, multi‐site, longitudinal community‐based research study: Canadian HIV Women's Sexual and Reproductive Health Cohort Study (CHIWOS). CHIWOS involved WLHIV in Ontario, British Columbia and Quebec, described in detail elsewhere 42, 43. CHIWOS baseline data (Time 1) includes 1422 women who completed the interview‐administered questionnaire between August 28, 2013 and May 1, 2015, and Time 2 data were collected between June 23, 2015 and January 31, 2017.

### 2.2 Participant recruitment and data collection

We used CHIWOS Time 1 and Time 2 data. WLHIV were recruited using non‐random, purposive sampling techniques such as Peer Research Associates (PRAs), word of mouth, HIV clinics, AIDS Service Organizations, Community Based Organizations, provincial CHIWOS Community Advisory Board networks, CHIWOS National Steering Committee networks, listservs, CHIWOS website, Facebook and Twitter. Individuals who self‐reported as living with HIV and identified as cisgender or transgender women 16 years and older were eligible for participation. Detailed information regarding recruitment strategies is reported elsewhere 44.

Our community‐based research approach involved recruiting and training 38 WLHIV as PRAs 45. PRAs recruited most participants and administered the interview to participants as a way of improving inclusion of diverse, marginalized populations in research, as well as to minimize the social distance between researchers and participants 45. The survey was conducted in English or French using online FluidSurveys™ at baseline and took approximately two hours to complete (median: 120 minutes, interquartile range: 90‐150). Participants in rural settings had the option to complete questionnaires by phone or Skype. Study participants provided informed consent and received a $50 CDN honorarium. Participants were given the option to opt out of the violence section of the questionnaire, complete the section themselves, or continue with the PRA. For this analysis, women were excluded if they chose not to answer the violence section in the survey.

### 2.3 Ethics

Research ethics board approval was granted from: Women's College Hospital, Simon Fraser University, University of British Columbia/Providence Health, and McGill University Health Centre. Prior to starting enrolment, study sites that had independent Research Ethics Boards received their own approval. Participants provided written informed consent or oral consent with a study team member as witness for phone/Skype questionnaires.

### 2.4 Measures

#### 2.4.1 Primary outcome: depression

Depression symptoms were assessed as a continuous variable using the Center for Epidemiologic Studies Depression Scale (CES‐D10) (range = 0‐30, Cronbach's α = 0.87) 46, 47.

#### 2.4.2 Explanatory variables

HIV‐related stigma was assessed using the HIV Stigma Scale‐Short Form (range = 0‐100, Cronbach's α = 0.85) 48, 49. Racial discrimination was measured using the Everyday Discrimination Scale‐Racism (range 8‐48, Cronbach's α = 0.96), and gender discrimination with the Everyday Discrimination Scale‐Sexism (range 8‐48, Cronbach's α = 0.94) 50, 51. The Everyday Discrimination Scales we used included 8 of the original 9 items; the item “You are called names or insulted” was not included due to pilot testing feedback that it overlapped with other stigma and violence items.

Recent violence experiences included the number of self‐reported (yes/no) experiences of violence (physical violence, verbal violence, control, sexual violence) in the past three months (range 0‐4) with the question “have any of the above experiences happened to you in the last three months?”. The recent violence variable was the sum of these reported experiences of violence.

#### 2.4.3 Socio‐demographics

Socio‐demographic variables included: age, province of residence, ethnicity, education level, and personal gross yearly income.

### 2.5 Data analysis

We calculated summary statistics of socio‐demographic, psychosocial and clinical variables, using means and standard deviation (SD) for continuous variables and frequencies and proportions for categorical variables. We examined the data using ANOVA for continuous variables and chi‐square or Fisher's exact test for categorical variables to examine the differences by recent violence experienced across socio‐demographic variables. Unadjusted and adjusted multifactorial linear regression analyses were examined to determine factors associated with depression.

There is no standard approach to measure intersectional stigma 52, 53. Davis 54 argues in the 2008 “Intersectionality as Buzzword” article that it may be in part due to its non‐prescriptive nature that the concept of intersectionality has been so widely taken up and applied across disciplines and methodological approaches. There are calls for innovation in quantitative intersectional stigma measurement 52, using strategies such as structural equation modelling (SEM) that has been applied in prior intersectional stigma research 26, 30, 31, 32. SEM allows the assessment of correlations between variables, including multiple forms of stigma.

We first conducted SEM using the maximum likelihood estimation method to examine the direct and indirect effect of HIV‐related stigma, racial discrimination and gender discrimination on depression and the mediation effect of recent violence. Model fit was assessed using: root‐mean‐square error of approximation (RMSEA), and comparative fit index (CFI). A score of <0.08 for RMSEA and a score >0.90 for CFI indicate an acceptable fit.

Following this, we used Time 1 and Time 2 data to conducted mixed‐effects regression to examine the relationship between HIV‐related stigma and depression over time, taking into consideration the effect of recent violence experiences. Mixed‐effects models are useful to understand the trajectory of changes accounting for within‐person and across‐person variability. In particular, fixed effects were used for years of the study (with a value of 1 assigned to Time 1 and 2 to Time 2), HIV‐related stigma and recent violence experience (0 = no recent violence experience; 1 = any form of violence in the past three months). We also controlled for covariates, including age at baseline assessment, ethno‐racial background, education level, province of residence and personal annual income at both Time 1 and Time 2.

A second SEM was then conducted using path analysis to investigate the cross‐lagged relationships between HIV‐related stigma, recent violence experiences and depression. The final model used the following procedures: (1) socio‐demographic factors (including age, ethno‐racial background, education level, province of residence, personal annual income) were treated as exogenous variables influencing the endogenous measures at Time 1; (2) the relationship among the endogenous variables at Time 1 (HIV‐related stigma, recent violence experience and depression) were assessed (e.g. HIV‐related stigma → recent violence experience → depression); (3) the endogenous variables at Time 1 (HIV‐related stigma, recent violence experiences and depression) were assessed for their longitudinal relationships across the two waves, with Time 1 variables used to predict Time 2 variables; (4) a full model added the following cross‐lagged effects: each Time 1 endogenous variable was considered to influence each of the Time 2 variables (e.g. Time 1 HIV‐related stigma → Time 2 recent violence experience → Time 2 depression).

Age (continuous), ethnicity (Indigenous, African, Caribbean, or Black (ACB), other vs. White), education level (high school or higher vs. less than high school), province (Ontario, Quebec vs. British Columbia) and personal annual income (>$40,000, $20,000‐40,000 vs. <$20,000) were controlled for to reduce their confounding effect. Two‐sided statistical tests were conducted with a significance level of 0.05. Statistical analyses were conducted using SAS (SAS, NC, USA) version 9.4 and Mplus (Mplus, CA, USA) and STATA 14.0 (STATA, TX, USA).

## 3 Results

### 3.1 Prevalence of recent violence

Of the total 1422 participants, 104 were excluded as they opted to skip the violent experiences section of the survey and 22 participants were excluded due to missing data related to adulthood violence. A total of 1296 participants were included in analyses of Time 1 data. The mean age was 42.8 years (SD = 10.7). Twenty‐two percent of the sample was Indigenous, 28% were African, Caribbean and Black, 43% were White and 8% were other ethnicities.

Table 1 displays socio‐demographic factors associated with recent violence, including no recent violence, one type of recent violence and multiple types of recent violence at Time 1 (described as +1 type of violence experience). One‐fifth (22%, n = 282) of participants reported experiencing recent violence (in the past three months), of which 54% (n = 152) reported experiencing one type of recent violence and 46% (n = 130) more than one type of recent violence. We also found that age, ethnicity (Indigenous vs. ACB or White or other), sexual orientation (lesbian, gay, bisexual, transgender, queer vs. heterosexual), having children vs. no children, lower education level, lower personal annual income, and providence of residence (British Columbia vs. Ontario or Quebec) were associated with increased likelihood of reporting recent violence at Time 1. During the follow‐up survey at Time 2, the total sample size was 1161. One‐quarter (25%, n = 286) of participants reported experiencing recent violence in the past three months, of which 73% (n = 210) reported experiencing one type of violence and 27% (n = 76) more than one type of violence.

**Table 1:** Socio‐demographic factors and number of types of violence^a^ in the past three months among women living with HIV in Canada at Time 1 (n=1296)

Sociodemographic Factor	Total N	Overall		No violence experience		1 type of violence experience		+1 type of violence experience		*p* Value
		(N=1296)		(N=1014, 78.24%)		(N=152, 11.73%)		(N=130, 10.03%)		
		N	(%)	N	(%)	N	(%)	N	(%)	
Age at interview date (years)	1296	Mean=42.79, SD=10.72	Range=16 to 74	Mean=43.21, SD=11.00	Range=16 to 74	Mean=41.47, SD=0.01	Range=18 to 72	Mean=41.00, SD=9.03	Range=18 to 67	<0.05
Age at interview by categories	1296									<0.05
<20 years old		12	(0.93)	7	(0.69)	3	(1.97)	2	(1.54)	
20 to 29 years old		118	(9.10)	90	(8.88)	17	(11.18)	11	(8.46)	
30 to 39 years old		389	(30.02)	308	(30.37)	40	(26.32)	41	(31.54)	
40 to 49 years old		420	(32.41)	311	(30.67)	55	(36.18)	54	(41.54)	
>=50 years old		357	(27.55)	298	(29.39)	37	(24.34)	22	(16.92)	
Ethnicity	1296									<0.001
Indigenous		280	(22)	198	(20)	35	(23)	47	(36)	
African, Caribbean Black		367	(28)	311	(31)	35	(23)	21	(16)	
Caucasian		551	(43)	433	(43)	70	(46)	48	(37)	
Other		98	(8)	72	(7)	12	(8)	14	(11)	
Immigration status	1291									0.202
Canadian citizen		1058	(82)	816	(81)	128	(85)	114	(88)	
Immigrant/permanent resident		233	(18)	194	(19)	23	(15)	16	(12)	
Sexual orientation	1291									<0.001
Heterosexual		1123	(87)	900	(89)	130	(86)	93	(72)	
LGBQ		168	(13)	109	(11)	22	(14)	37	(28)	
Province interview conducted	1296									<0.01
BC		324	(25)	230	(23)	44	(29)	50	(38)	
ON		646	(50)	518	(51)	72	(47)	56	(43)	
QC		322	(25)	266	(26)	36	(24)	24	(19)	
Relationship status 1290										0.328
Married/common‐law		425	(33)	329	(33)	47	(31)	49	(39)	
Single		621	(48)	483	(48)	82	(54)	56	(44)	
Separated/Divorced/ widowed		244	(19)	199	(20)	23	(15)	22	(17)	
Education level	1296									<0.01
Elementary school		197	(15)	135	(13)	34	(22)	28	(22)	
High school		556	(43)	432	(43)	59	(39)	65	(50)	
Trade/technical/college		353	(27)	286	(28)	41	(27)	26	(20)	
Post‐grad/ undergraduate/other		190	(15)	161	(16)	18	(12)	11	(8)	
Personal gross yearly income	1267									<0.01
< $20000		904	(71)	681	(69)	121	(80)	102	(80)	
$20000‐$40000		227	(18)	191	(19)	16	(11)	20	(16)	
>$40000		136	(11)	117	(12)	14	(9)	5	(4)	
Personal gross yearly income at follow‐up	1124									0.064
< $20000		743	(66.10)	575	(64.17)	89	(71.77)	79	(75.96)	
$20000 to $40000		263	(23.40)	219	(24.44)	24	(19.35)	20	(19.23)	
>$40000		118	(10.50)	102	(11.38)	11	(8.87)	5	(4.81)	
HIV‐related stigma at baseline	1282	Mean=57.10, SD=19.10	Range=0 to 100	Mean=55.89, SD=19.52	Range=0 to 100	Mean=57.13, SD=19.99	Range=17.5 to 100	Mean=66.45, SD=19.81	Range=10 to 100	<0.001
HIV‐related stigma at follow‐up	1235	Mean=57.48, SD=18.94	Range=0 to 100	Mean=56.66, SD=18.81	Range=0 to 100	Mean=58.72, SD=18.68	Range=0 to 100	Mean=64.37, SD=20.08	Range=15 to 100	<0.001
Gender discrimination at baseline	1278	Mean=19.46, SD=9.88	Range=8 to 48	Mean=18.22, SD=9.42	Range=8 to 47	Mean=22.44, SD=9.57	Range=8 to 48	Mean=25.69, SD=10.67	Range=8 to 48	<0.001
Racial discrimination at baseline	1278	Mean=18.76, SD=10.88	Range=8 to 48	Mean=17.67, SD=10.23	Range=8 to 48	Mean=21.18, SD=11.60	Range=8 to 48	Mean=24.27, SD=12.68	Range=8 to 48	<0.001
Depression at baseline	1252	Mean=9.91, SD=7.53	Range=0 to 30	Mean=8.76, SD=7.19	Range=0 to 30	Mean=12.27, SD=7.13	Range=0 to 30	Mean=16.10, SD=6.89	Range=2 to 30	<0.001
Depression at follow‐up	1188	Mean=9.26, SD=7.73	Range=0 to 30	Mean=7.92, SD=7.11	Range=0 to 30	Mean=12.82, SD=7.73	Range=0 to 30	Mean=16.70, SD=8.20	Range=0 to 30	<0.001

Note: 1. *p*‐values were calculated using ANOVA or Chi square test. ACB, African, Caribbean, or Black; LGBTQ, lesbian, gay, bisexual, transgender, queer; +1 type of violence experience refers to experiencing >1 type of violence.

Types of violence included verbal, physical, sexual and control violence

### 3.2 Factors associated with depression at Time 1 and Time 2

Table 2 summarizes the linear regression coefficients of factors associated with depression during Time 1 and Time 2. In adjusted analyses controlling for socio‐demographic factors (age, ethnicity, education level, province and income), higher HIV‐related stigma (Acoef: 0.07, 95% CI: 0.05‐0.10, *p* < 0.001), gender discrimination (Acoef: 0.12, 95% CI: 0.06‐0.18, *p* < 0.001), and recent violence experiences (Acoef: 2.21, 95% CI: 1.71‐2.70) were associated with higher levels of depression. At Time 2, higher levels of depression were associated with HIV‐related stigma (Acoef: 0.04, 95% CI: 0.02‐0.06) and recent violence experiences (Acoef: 2.44, 95% CI: 1.95‐2.92), adjusting for socio‐demographic factors.

**Table 2:** Unadjusted and adjusted regression analyses on stigma and recent violence experiences associated with depression at Time 1 and Time 2 (N = 1296)

Variables	Depression at baseline		Depression at follow‐up	
	Unadjusted Ceof (95% CI)	Adjusted Ceof (95% CI)	Unadjusted Ceof (95% CI)	Adjusted Ceof (95% CI)
HIV‐related stigma	0.09 (0.08‐0.12)*	0.07 (0.05‐0.10)*	0.05 (0.04‐0.07)*	0.04 (0.02‐0.06)*
Racial discrimination	0.13 (0.10‐0.17)*	−0.01 (−0.07‐0.05)	N/A	N/A
Gender discrimination	0.20 (0.16‐0.24)*	0.12 (0.06‐0.18)*	N/A	N/A
Number of violence experiences in the past three months	2.83 (2.33‐3.32)*	2.21 (1.71‐2.70)*	2.99 (2.52‐3.46)*	2.44 (1.95‐2.92)*

Covariates: age, ethnicity, education level, province, and personal annual income.

*p < 0.001.

### 3.3 SEM results for Time 1 data

**Figure 1:**
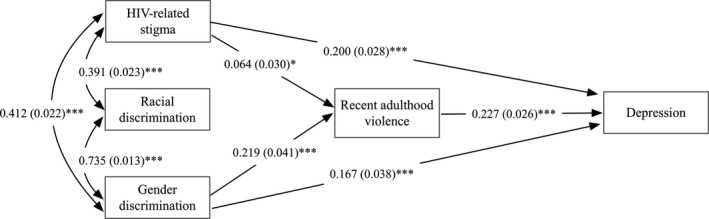
Mediational model of experiencing recent violence in the past three months among women living with HIV in Canada at baseline. Recent adulthood violence: violence experienced in past three months. *p < 0.05, ***p < 0.001.

The first SEM (Figure 1) was tested to assess the direct and indirect effects of HIV‐related stigma, racial discrimination and gender discrimination on depression and the mediation effect of recent violence experiences at Time 1. Final model fit indices suggest that the model fit the data well (CFI = 1.000; RMSEA = 0.000 (90% CI: 0.000, 0.000)). Figure 1 illustrates the model with standard coefficients and the significance levels of each pathway. The standardized coefficient indicated that with a SD increase of the independent variable, the dependent variable would increase by x SD, holding all other variables constant. Standard errors were included in parenthesis.

Table 3 details the path analysis findings regarding pathways from stigma to depression, via recent violence experiences. The direct path from HIV‐related stigma (direct effect: β = 0.200, *p* < 0.001; indirect effect: β = 0.014, *p* < 0.05) to depression was significant, and recent violence accounted for 6.5% (0.014/0.215) of the total effect. Gender discrimination had a significant direct and indirect effect on depression (direct effect: β = 0.167, *p* < 0.001; indirect effect: β = 0.050, *p* < 0.001), and recent violence explained 23.15% of the total effect. Racial discrimination did not have a significant direct or indirect effect on depression.

**Table 3:** Final path analysis of depression via experiences of stigma and recent violence experiences at Time 1 (N = 1296)

Parameter	Coefficient (SE)	Critical ratio	*p*	Standardized estimate
Depression ON				
HIV‐related stigma	0.075 (0.010)	7.173	<0.001	0.200
Gender discrimination	0.124 (0.029)	4.348	<0.001	0.167
Racial discrimination	−0.010 (0.029)	−0.030	0.976	−0.001
Number of recent violence	2.210 (0.249)	8.441	<0.001	0.227
Number of recent violence ON				
HIV‐related stigma	0.003 (0.001)	2.091	<0.05	0.064
Gender discrimination	0.018 (0.003)	5.255	<0.001	0.219
Racial discrimination	0.001 (0.002)	0.021	0.263	0.001
HIV‐related stigma WITH				
Gender discrimination	71.414 (5.267)	12.692	<0.001	0.412
Racial discrimination	74.319 (6.396)	11.632	<0.001	0.391
Gender discrimination WITH				
Racial discrimination	82.065 (5.329)	15.399	<0.001	0.735

Covariates: age, ethnicity, education level, province, and personal annual income.

SE, standard error.

### 3.4 Mixed‐effects modelling and SEM results for Time 1 and Time 2 data

**Figure 2:**
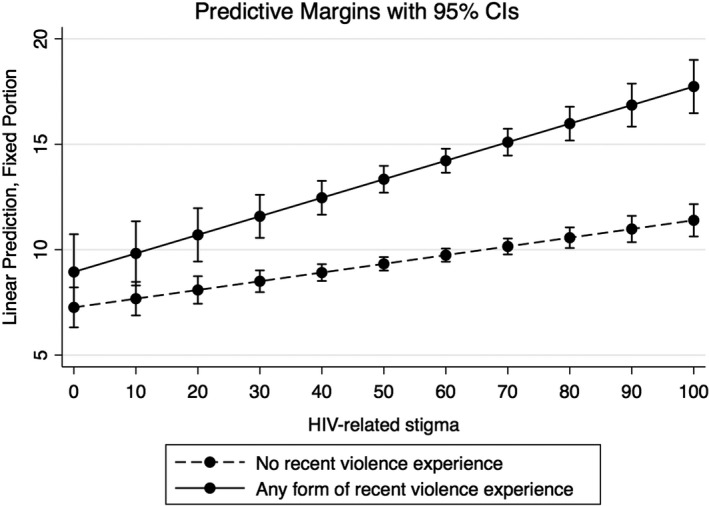
Predicted marginalized effects of recent violence experiences with HIV‐related stigma on depression. Recent adulthood violence: violence experienced in the past three months.

Mixed‐effects regression results (Table 4) indicated a significant positive relationship over time between HIV‐related stigma and depression scores (Acoef: 0.04, 95% CI: 0.03, 0.06, *p* < 0.001), and recent violence experiences (any vs. none) and depression scores (Acoef: 1.95, 95% CI: 0.29, 4.42, *p* < 0.05), controlling for age, ethnicity, education, personal annual income and province of residency. There was also a significant interaction between HIV‐related stigma and any recent violence experiences with depression scores (Acoef: 0.04, 95% CI: 0.01, 0.07, *p* < 0.05) over time. Recent violence experiences also moderated the relationship between HIV‐related stigma and depression. As illustrated in Figure 2, recent violence experiences exacerbated the impact of HIV‐related stigma on depression. Specifically, with no experiences of recent violence and the lowest HIV‐related stigma score (0), the predicted mean CES‐D score was 7. However, if a participant reported recent violence experiences and the highest HIV‐related stigma score (100), the predicted mean CES‐D score was 18.

**Table 4:** Mixed effects modelling of the associations between HIV‐related stigma, experiences of recent violence, and depression over time for women living with HIV in Canada (n = 1161)

Depression	Coefficient/beta (95% CI)	SE	*p*
HIV‐related stigma	0.04 (0.03‐0.06)	0.01	<0.001
Recent violence experience (any vs. none)	1.95 (0.29‐4.42)	1.05	<0.05
HIV‐related stigma × any form of recent violence experience	0.04 (0.01‐0.07)	0.02	<0.05

Covariates: age, ethnicity, education level, province, and personal annual income.

SE, standard error.

Table 5 provides the final path analysis results of associations between HIV‐related stigma, recent violence experiences, and depression over time. Final model fit indices suggest that the model fit the data well (CFI = 0.984; RMSEA = 0.043 (90% CI: 0.024, 0.063), Standardized Root Mean Square Residual (SRMR) = 0.030).

**Table 5:** Final path analysis of HIV‐related stigma on depression via recent violence experiences over time (N = 1161)

Parameter	Coefficient (SE)	Critical ratio	*p*	Standardized estimate (SE)
Depression (Time 2) ON				
HIV‐related stigma (Time 2)	0.018 (0.009)	2.12	<0.05	0.059 (0.028)
Recent violence experiences (Time 2)	1.821 (0.256)	7.11	<0.001	0.219 (0.029)
Depression (Time 1)	0.291 (0.023)	12.88	<0.001	0.377 (0.028)
Recent violence experiences (Time 1)	0.470 (0.221)	2.12	<0.05	0.064 (0.031)
HIV‐related stigma (Time 1)	−0.004 (0.010)	−0.41	0.684	−0.014 (0.034)
Recent violence experiences (Time 2) ON				
HIV‐related stigma (Time 2)	0.002 (0.001)	1.92	0.055	0.067 (0.035)
Depression (Time 1)	0.011 (0.003)	4.42	<0.001	0.129 (0.031)
Recent violence experiences (Time 1)	0.254 (0.026)	9.65	<0.001	0.302 (0.030)
HIV‐related stigma (Time 1)	−0.001 (0.001)	−0.94	0.346	−0.035 (0.037)
HIV‐related stigma (Time 2) ON				
Depression (Time 1)	−0.004 (0.069)	−0.06	0.951	−0.002 (0.028)
Recent violence experiences (Time 1)	0.834 (0.647)	1.29	0.198	0.035 (0.027)
HIV‐related stigma (Time 1)	0.551 (0.025)	22.89	<0.001	0.592 (0.019)
Depression (Time 1) ON				
HIV‐related stigma (Time 1)	0.066 (0.011)	5.95	<0.001	0.178 (0.029)
Recent violence experiences (Time 1)	2.765 (0.284)	9.73	<0.001	0.291 (0.029)
Recent violence experiences (Time 1) ON				
HIV‐related stigma (Time 1)	0.005 (0.001)	4.41	<0.001	0.138 (0.031)

Covariates: age, ethnicity, education level, province, and personal annual income.

SE, standard error.

**Figure 3:**
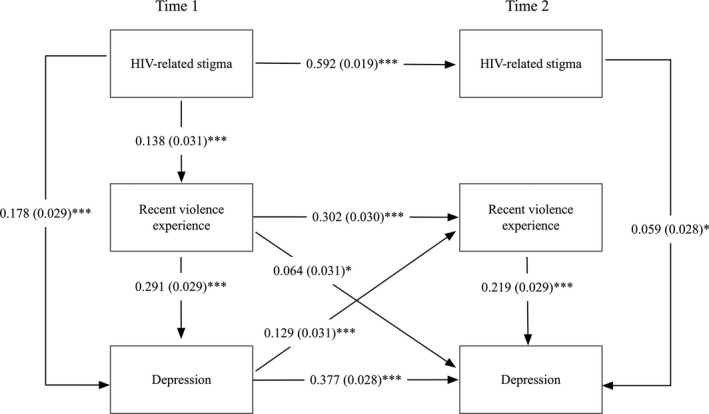
Longitudinal mediational model of experiencing recent violence in the past three months among women living with HIV in Canada. Recent adulthood violence: violence experienced in the past three months. *p < 0.05, ***p < 0.001.

At Time 1 depression was positively associated with HIV‐related stigma and recent violence experiences. HIV‐related stigma had both significant direct and indirect effects on depression (direct effect: β = 0.178, *p* < 0.001; indirect effect: β = 0.040, *p* < 0.001), with recent violence experiences accounting for 51% (0.291/0.510) of the total effect. From Time 1 to Time 2, as presented in Figure 3, HIV‐related stigma at Time 1 was associated with HIV‐related stigma at Time 2; recent violence experiences and depression at Time 1 were associated with recent violence experiences at Time 2; and recent violence experiences and depression at Time 1 were associated with depression at Time 2. HIV‐related stigma at Time 2 and recent violence experiences at Time 2 were also associated with depression at Time 2.

## 4 Discussion

Over one‐fifth of this Canadian cohort of WLHIV experienced violence in the past three months at Time 1, and one‐quarter at Time 2, indicating an ongoing issue of violence that requires urgent focus. HIV‐related stigma and gender discrimination were associated with recent violence as well as depression at baseline. Over time, recent violence experiences interacted with HIV‐related stigma with harmful effects on depression.

The cross‐sectional model of recent violence at Time 1 suggests that both HIV‐related stigma and gender discrimination have direct and indirect effects on depression via recent violence. The longitudinal model suggests that HIV‐related stigma at Time 1 is associated with HIV‐related stigma at Time 2, and HIV‐related stigma at Time 2 remains directly and indirectly associated with depression via recent violence. We also establish connections between (1) HIV‐related stigma and exposure to violence at Time 1, (2) recent violence and depression at Time 1 with recent violence at Time 2, and (3) recent violence and depression at Time 1, and recent violence at Time 2, with depression at Time 2. Both models indicate accumulating health risks of stigma, recent violence and depression among WLHIV, aligning with the call to include larger social forces (such as stigma) in syndemics analyses and to test a serially causal epidemic model 39. The interaction we identified between HIV‐related stigma and violence over time that exacerbates their effects on depression suggests the possibility of “synergistically interacting epidemics” 39 of stigma and violence among WLHIV.

Our findings also align with a systematic review of longitudinal effects of recent IPV on depression among women, suggesting that WLHIV also experience the harmful impacts of violence exposure on depression 15. Similar to these findings, we also found a bidirectional relationship between recent violence and depression over time. The causal pathways between depression and recent IPV are understudied, but it is possible that depression symptoms reduce energy, hope, self‐efficacy and motivation to engage with social service programs to acquire assistance to leave violent relationships 15, 55. We add to this knowledge base by identifying HIV‐related stigma as an important cause of violence and depression among WLHIV that may present further barriers to leaving violent situations.

Research should further explore the role of racial discrimination in the lives of WLHIV. Racial discrimination was not significantly associated with violence or depression in multifactorial analysis yet was associated with depression in univariate analysis. This finding calls for further nuanced research on measuring racism, its health effects, and associated coping strategies among WLHIV in Canada 56. It also signals the need to consider and assess institutionalized racism that may be associated with stigma, violence and depression. For instance, Prather *et al*.'s 57 recent review of racism and African American women's health points to the need to explore health effects of racism in healthcare, criminal justice, housing, education and employment systems.

Violence experiences among participants varied by socio‐demographic characteristics. Higher levels of recent violence in British Columbia in comparison with Ontario and Quebec may be due to higher rates of violence against women 16 and the higher crime severity index 58 in the census metropolitan area of Vancouver in comparison with Toronto or Montreal. Rates of violence were found to be the highest among Indigenous women, which reflects previous research 59, 60 and national data 16. Indigenous women's vulnerability to violence and HIV is rooted in historical and ongoing trauma from colonization, residential schools and racism 59, 61. Violence was higher among those with lower income and education, corroborating previous research involving WLHIV 62. Low‐income women may rely on partners for food and housing stability, constraining their ability to leave IPV contexts 63, 64.

The study has limitations. The non‐random sampling limits generalizability. We may have included persons who were more connected to HIV care services. Longitudinal data on racial and gender discrimination could add complexity to the analyses of intersectional stigma. We did not assess the perpetrator of violence, and relationship to the perpetrator may play a role in psychological consequences of violence 65. Our racial and gender discrimination measures only assessed enacted stigma, precluding understanding the potential effects of perceived and internalized forms of racism and sexism. The Everyday Discrimination Scale was developed in the U.S. and could be further validated with racialized women in Canada. In this study we assessed gender discrimination, racial discrimination, and HIV‐related stigma separately. Future research could assess the simultaneous effects of intersectional stigma. Finally, data were self‐reported and may be vulnerable to social desirability bias.

## 5 Conclusions

Our findings advance knowledge regarding violence among WLHIV. To the best of our knowledge this is among the first longitudinal studies to examine connections between HIV‐related stigma, recent violence and depression among WLHIV. Findings suggest that both stigma and violence must be addressed in order reduce depression among WLHIV, as the deleterious effects of stigma on depression may persist, even if violence is reduced. This has implications for theoretical and methodological innovations regarding intersectional stigma reduction, as a systematic review of stigma interventions for ACB WLHIV highlighted that none addressed more than one form of stigma 66. Along with others, we propose the use of women‐centred, trauma‐informed and violence‐aware practice that addresses PTSD, depression, intersectional stigma, incorporates harm reduction and ensures screening for all forms of violence in HIV and health care and support services for WLHIV 67, 68, 69, 70.

## Competing interests

The authors have no conflicts of interest to disclose.

## Authors’ contributions

CHL led writing and conceptualizing of the manuscript. YW conducted data analysis. NM substantially contributed to writing the manuscript. UA contributed to writing the manuscript. AK, PO, NO, ADP, MRL, MF, VN and TC contributed to conceptualizing the study, data collection and interpretation of findings. All authors provided edits and feedback and approval of the final version.

## Appendix 1

### 1.1

The CHIWOS Research Team would like to thank women living with HIV for their contributions to this study. We also thank the national team of co‐investigators, collaborators, and Peer Research Associates and acknowledge the national Steering Committee, our three provincial Community Advisory Boards, the National CHIWOS Aboriginal Advisory Board, the BC Centre for Excellence in HIV/AIDS for data support and analysis, and all our partnering organizations for supporting the study. Listed here are all research team members and affiliated institutions; all those not listed by name on the title page are to be hyperlinked as authors: The CHIWOS Research Team: British Columbia: Aranka Anema (University of British Columbia), Denise Becker (Positive Living Society of British Columbia), Lori Brotto (University of British Columbia), Allison Carter (British Columbia Centre for Excellence in HIV/AIDS and Simon Fraser University), Claudette Cardinal (Simon Fraser University), Guillaume Colley (British Columbia Centre for Excellence in HIV/AIDS), Erin Ding (British Columbia Centre for Excellence), Janice Duddy (Pacific AIDS Network), Nada Gataric (British Columbia Centre for Excellence in HIV/AIDS), Robert S Hogg (British Columbia Centre for Excellence in HIV/AIDS and Simon Fraser University), Terry Howard (Positive Living Society of British Columbia), Shahab Jabbari (British Columbia Centre for Excellence), Evin Jones (Pacific AIDS Network), Mary Kestler (Oak Tree Clinic, BC Women's Hospital and Health Centre), Andrea Langlois (Pacific AIDS Network), Viviane Lima (British Columbia Centre for Excellence in HIV/AIDS), Elisa Lloyd‐Smith (Providence Health Care), Melissa Medjuck (Positive Women's Network), Cari Miller (Simon Fraser University), Deborah Money (Women's Health Research Institute), Valerie Nicholson (Simon Fraser University), Gina Ogilvie (British Columbia Centre for Disease Control), Sophie Patterson (Simon Fraser University), Neora Pick (Oak Tree Clinic, BC Women's Hospital and Health Centre), Eric Roth (University of Victoria), Kate Salters (Simon Fraser University), Margarite Sanchez (ViVA, Positive Living Society of British Columbia), Jacquie Sas (CIHR Canadian HIV Trials Network), Paul Sereda (British Columbia Centre for Excellence in HIV/AIDS), Marcie Summers (Positive Women's Network), Christina Tom (Simon Fraser University, British Columbia), Lu Wang (British Columbia Centre for Excellence), Kath Webster (Simon Fraser University), Wendy Zhang (British Columbia Centre for Excellence in HIV/AIDS). Ontario: Rahma Abdul‐Noor (Women's College Research Institute), Jonathan Angel (Ottawa Hospital Research Institute), Fatimatou Barry (Women's College Research Institute), Greta Bauer (University of Western Ontario), Kerrigan Beaver (Women's College Research Institute), Anita Benoit (Women's College Research Institute), Breklyn Bertozzi (Women's College Research Institute), Sheila Borton (Women's College Research Institute), Tammy Bourque (Women's College Research Institute), Jason Brophy (Children's Hospital of Eastern Ontario), Ann Burchell (Ontario HIV Treatment Network), Allison Carlson (Women's College Research Institute), Lynne Cioppa (Women's College Research Institute), Jeffrey Cohen (Windsor Regional Hospital), Tracey Conway (Women's College Research Institute), Curtis Cooper (Ottawa Hospital Research Institute), Jasmine Cotnam (Women's College Research Institute), Janette Cousineau (Women's College Research Institute), Annette Fraleigh (Women's College Research Institute), Brenda Gagnier (Women's College Research Institute), Claudine Gasingirwa (Women's College Research Institute), Saara Greene (McMaster University), Trevor Hart (Ryerson University), Shazia Islam (Women's College Research Institute), Charu Kaushic (McMaster University), Logan Kennedy (Women's College Research Institute), Desiree Kerr (Women's College Research Institute), Maxime Kiboyogo (McGill University Health Centre), Gladys Kwaramba (Women's College Research Institute), Lynne Leonard (University of Ottawa), Johanna Lewis (Women's College Research Institute), Carmen Logie (University of Toronto), Shari Margolese (Women's College Research Institute), Marvelous Muchenje (Women's Health in Women's Hands), Mary (Muthoni) Ndung'u (Women's College Research Institute), Kelly O'Brien (University of Toronto), Charlene Ouellette (Women's College Research Institute), Jeff Powis (Toronto East General Hospital), Corinna Quan (Windsor Regional Hospital), Janet Raboud (Ontario HIV Treatment Network), Anita Rachlis (Sunnybrook Health Science Centre), Edward Ralph (St. Joseph's Health Care), Sean Rourke (Ontario HIV Treatment Network), Sergio Rueda (Centre for Addiction and Mental Health (CAMH)), Roger Sandre (Haven Clinic), Fiona Smaill (McMaster University), Stephanie Smith (Women's College Research Institute), Tsitsi Tigere (Women's College Research Institute), Wangari Tharao (Women's Health in Women's Hands), Sharon Walmsley (Toronto General Research Institute), Wendy Wobeser (Kingston University), Jessica Yee (Native Youth Sexual Health Network), Mark Yudin (St‐Michael's Hospital). Quebec: Dada Mamvula Bakombo (McGill University Health Centre), Jean‐Guy Baril (Université de Montréal), Nora Butler Burke (University Concordia), Pierrette Clément (McGill University Health Center), Janice Dayle (McGill University Health Centre), Danièle Dubuc (McGill University Health Centre), Mylène Fernet (Université du Québec à Montréal), Danielle Groleau (McGill University), Aurélie Hot (COCQ‐SIDA), Marina Klein (McGill University Health Centre), Carrie Martin (Native Women's Shelter of Montreal), Lyne Massie (Université de Québec à Montréal), Brigitte Ménard (McGill University Health Centre), Nadia O'Brien (McGill University Health Centre and Université de Montréal), Joanne Otis (Université du Québec à Montréal), Doris Peltier (Canadian Aboriginal AIDS Network), Alie Pierre (McGill University Health Centre), Karène Proulx‐Boucher (McGill University Health Centre), Danielle Rouleau (Centre Hospitalier de l'Université de Montréal), Édénia Savoie (McGill University Health Centre), Cécile Tremblay (Centre Hospitalier de l'Université de Montréal), Benoit Trottier (Clinique l'Actuel), Sylvie Trottier (Centre Hospitalier Universitaire de Québec), Christos Tsoukas (McGill University Health Centre). Other Canadian provinces or international jurisdictions: Jacqueline Gahagan (Dalhousie University), Catherine Hankins (University of Amsterdam), Renee Masching (Canadian Aboriginal AIDS Network), Susanna Ogunnaike‐Cooke (Public Health Agency of Canada). All other CHIWOS Research Team Members who wish to remain anonymous.
